# Inhibition of succinate dehydrogenase by the mitochondrial chaperone TRAP1 has anti-oxidant and anti-apoptotic effects on tumor cells

**DOI:** 10.18632/oncotarget.2472

**Published:** 2014-11-15

**Authors:** Giulia Guzzo, Marco Sciacovelli, Paolo Bernardi, Andrea Rasola

**Affiliations:** ^1^ CNR Neuroscience Institute and Department of Biomedical Sciences, University of Padova, 35121 Padova, Italy; ^2^ Medical Research Council Cancer Unit, University of Cambridge, Hutchison/MRC Research Centre, Cambridge, United Kingdom

**Keywords:** TRAP1, mitochondria, chaperones, cancer metabolism, ROS, succinate dehydrogenase

## Abstract

TRAP1 is a mitochondrial chaperone highly expressed in many tumor types; it inhibits respiratory complex II, down-modulating its succinate dehydrogenase (SDH) enzymatic activity. SDH inhibition in turn leads to a pseudohypoxic state caused by succinate-dependent HIF1α stabilization and promotes neoplastic growth. Here we report that TRAP1 inhibition of SDH also shields cells from oxidative insults and from the ensuing lethal opening of the mitochondrial permeability transition pore. This anti-oxidant activity of TRAP1 protects tumor cells from death in conditions of nutrient paucity that mimic those encountered in the neoplasm during the process of malignant accrual, and it is required for *in vitro* tumorigenic growth. Our findings demonstrate that SDH inhibition by TRAP1 is oncogenic not only by inducing pseudohypoxia, but also by protecting tumor cells from oxidative stress.

## INTRODUCTION

Cancer cells are exposed to conditions that increase their reactive oxygen species (ROS) levels, such as abnormal activation of signal transduction pathways, rewiring of metabolic machinery and aberrant environmental inputs [1–3]. Hence, to avoid the detrimental effects of oxidative stress, cells must rapidly implement their anti-oxidant defenses to set a novel homeostatic redox equilibrium in early neoplastic phases, in order to adapt to metabolic and signalling changes that unbalance their redox control systems [1, 4]. Indeed, it was shown [5] that several oncogenes induce a ROS scavenging program that is required for tumor initiation and to escape cell death caused by unrestrained oxidative insults. A key effector of ROS-induced cell death is the permeability transition pore (PTP), a mitochondrial channel extremely sensitive to oxidants whose opening rapidly elicits irreversible mitochondrial damage and cell demise [6–8]. It was shown that oncogenic signals lead to PTP inhibition, which contributes to the capability of cancer cells to survive under several stress conditions [9–11].

However, high ROS levels can also contribute to the neoplastic process in several ways, e.g. by modulating pro-neoplastic signalling pathways and increasing genetic instability of tumor cells, their proliferation rate, resistance to stress conditions and invasive and metastatic properties [12, 13]. Mutations caused by oxidants can induce more metabolic changes and ROS generation, in a feed-forward loop that strongly enhances tumor aggressiveness. It is reasonable to envision that redox conditions might fluctuate during the complex process of tumor progression, and that a shift towards higher intracellular ROS levels may become extremely important at advanced stages of tumor growth, when increasing mutagenicity is essential to shape malignant properties of neoplasms [1, 4]. A postulate of this multifaceted role of ROS in neoplastic transformation is that malignant cells are endowed with an extremely fragile redox balance, which renders them vulnerable to oxidants. Therefore, a detailed comprehension of the mechanisms of ROS control that occur in tumorigenesis could be instrumental for the development of selective anti-neoplastic compounds [2].

One of the main sources of intracellular ROS is mitochondrial respiration, and its down-modulation during neoplastic transformation can strongly impact on mitochondrial superoxide levels [3, 4, 14]. We have recently shown that the mitochondrial chaperone TRAP1, which is highly expressed in a variety of tumor cell types [15], decreases the succinate dehydrogenase (SDH) enzymatic activity of respiratory complex II, thus inhibiting respiration. This mode of respiratory regulation has oncogenic implications, as the accumulation of succinate elicited by SDH inhibition stabilizes the transcription factor HIF1α and prompts tumor growth [16]. Accordingly, loss of function SDH mutations cause the hereditary paraganglioma/phaeochromocytoma syndrome and are associated with some cases of gastrointestinal stromal tumors or renal tumors; in all these neoplasms, succinate-dependent stabilization of HIF1α is believed to play a key pro-neoplastic role [17, 18].

SDH is an important site of ROS generation [19–21]. After succinate oxidation to fumarate, which occurs in SDHA subunit, electrons are funneled through Fe/S sites in SDHB subunit to coenzyme Q binding sites formed by SDHB/C/D proteins, leading to ubiquinone reduction and the ensuing electron flow to respiratory complex III [22]. Any interference in this chain of redox reactions results in leakage of electrons and superoxide formation following interaction with molecular oxygen. Indeed, several molecules that target the ubiquinone binding site of SDH eliciting superoxide generation cause apoptosis of cancer cells and are currently under study as potential chemotherapeutics [23]. Conversely, upstream impairment of succinate oxidation by SDHA subunit can abrogate any electron flow to further SDH components, thus inhibiting ROS generation.

Therefore, inactivating SDH mutations in specific cancer subsets, or down-regulation of SDH activity by TRAP1 in a wider number of tumor types, might contribute to the neoplastic process not only by stabilizing HIF1, but also by changing the redox equilibrium of malignant cells [15, 17, 23]. Indeed, it was reported that TRAP1 exerts anti-oxidant functions, but the molecular mechanisms of this regulation have remained poorly characterized [24–26]. Moreover, it was unclear whether keeping ROS at low levels might contribute to the tumorigenic function of TRAP1 [15]. Here we find that the anti-oxidant activity of TRAP1 is caused by its inhibitory interaction with SDH. The ensuing respiratory down-regulation abrogates ROS-dependent opening of the mitochondrial PTP, minimizing oxidative damage of tumor cells and promoting *in vitro* tumorigenesis.

## RESULTS

### TRAP1 protects from oxidative stress

**Figure 1:**
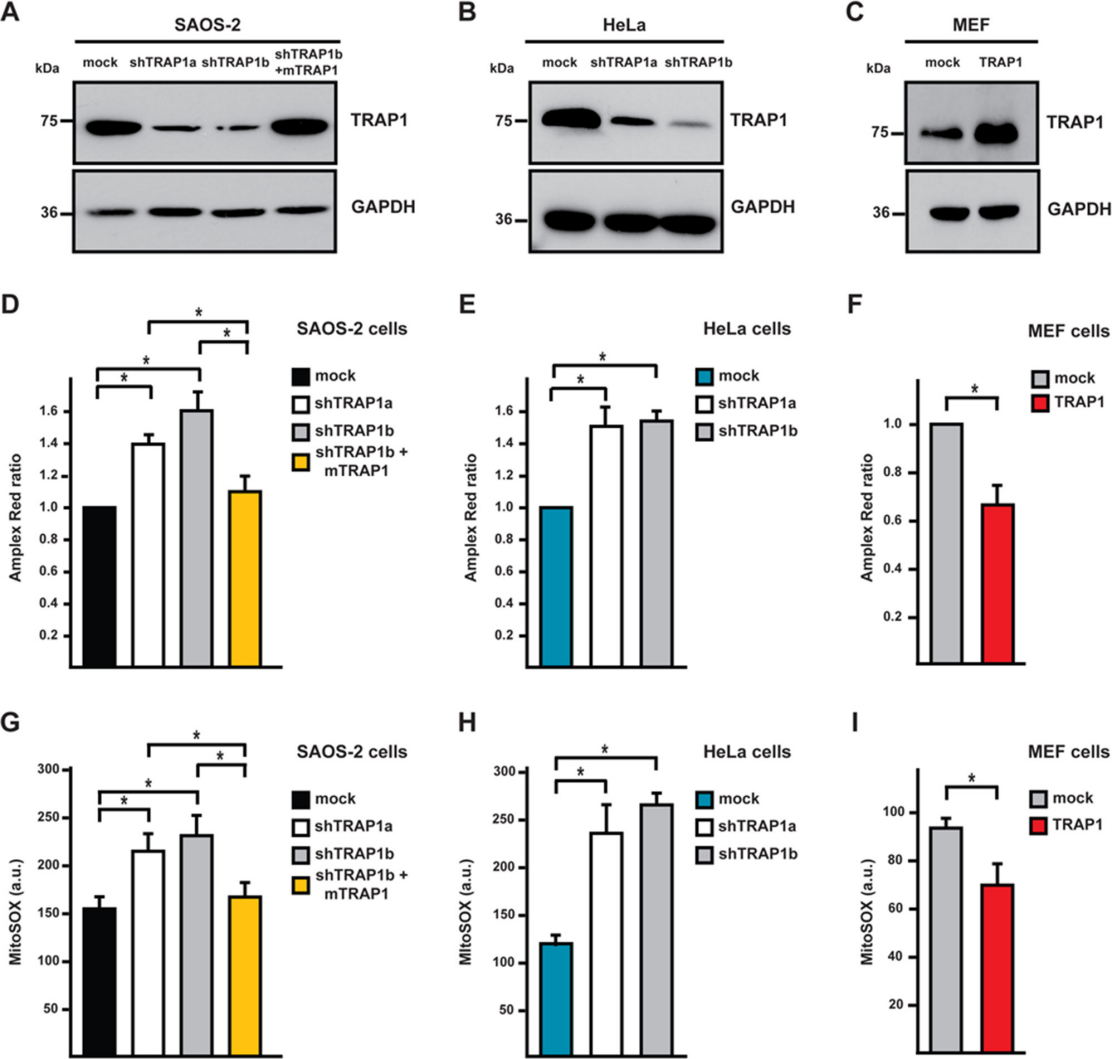
TRAP1 expression decreases intracellular ROS levels **(A-C)** TRAP1 expression levels were measured in SAOS-2 osteosarcoma cells (A), HeLa cervix carcinoma cells (B) and mouse embryo fibroblasts (MEF; C) by Western immunoblot assays. GAPDH was used as a loading control. SAOS-2 and Hela cells were transfected either with a scrambled shRNA (mock) or with two different TRAP1 shRNAs (shTRAP1a and shTRAP1b); shTRAP1b cells transfected with a mouse TRAP1 cDNA insensitive to interference by shTRAP1 were indicated as shTRAP1b + mTRAP1. MEFs were stably transfected with either a TRAP1 cDNA or with an empty vector (mock). **(D-F)** Intracellular hydrogen peroxide levels were assessed by Amplex Red in lysates from SAOS-2 (D), HeLa (E) or MEF (F) cells. **(G-I)** Cytofluorimetric analysis of mitochondrial superoxide levels was carried out by staining SAOS-2 (G), HeLa (H) or MEF (I) cells with the fluorescent probe MitoSox. All data are reported as mean±SD values (n≥3; *P<0.05 in a Student's *t* test).

TRAP1 was reported to protect tumor cells from oxidative stress, even if the molecular mechanism remains unclear [24, 25, 27–29]. Accordingly, we found that knocking-down TRAP1 expression in human osteosarcoma SAOS-2 cells and human cervix carcinoma HeLa cells (Fig. 1A, 1B; cells expressing short hairpin TRAP1 RNAs were dubbed shTRAP1), caused a constitutive increase in the levels of intracellular ROS and of mitochondrial superoxide anion (Fig. 1D, 1E, 1G, 1H). Notably, when TRAP1 expression was reinstalled in shTRAP1 cells by using a mouse cDNA (which is insensitive to the human shTRAP1 RNA [16]; cells harboring this construct are indicated as mTRAP1), both global ROS and mitochondrial superoxide levels returned to basal values (Fig. 1D, 1G). Similarly, TRAP1 overexpression in non-transformed mouse embryo fibroblasts (MEFs; Fig. 1C) down-regulated intracellular ROS and mitochondrial superoxide anion levels (Fig. 1F, 1I).

**Figure 2:**
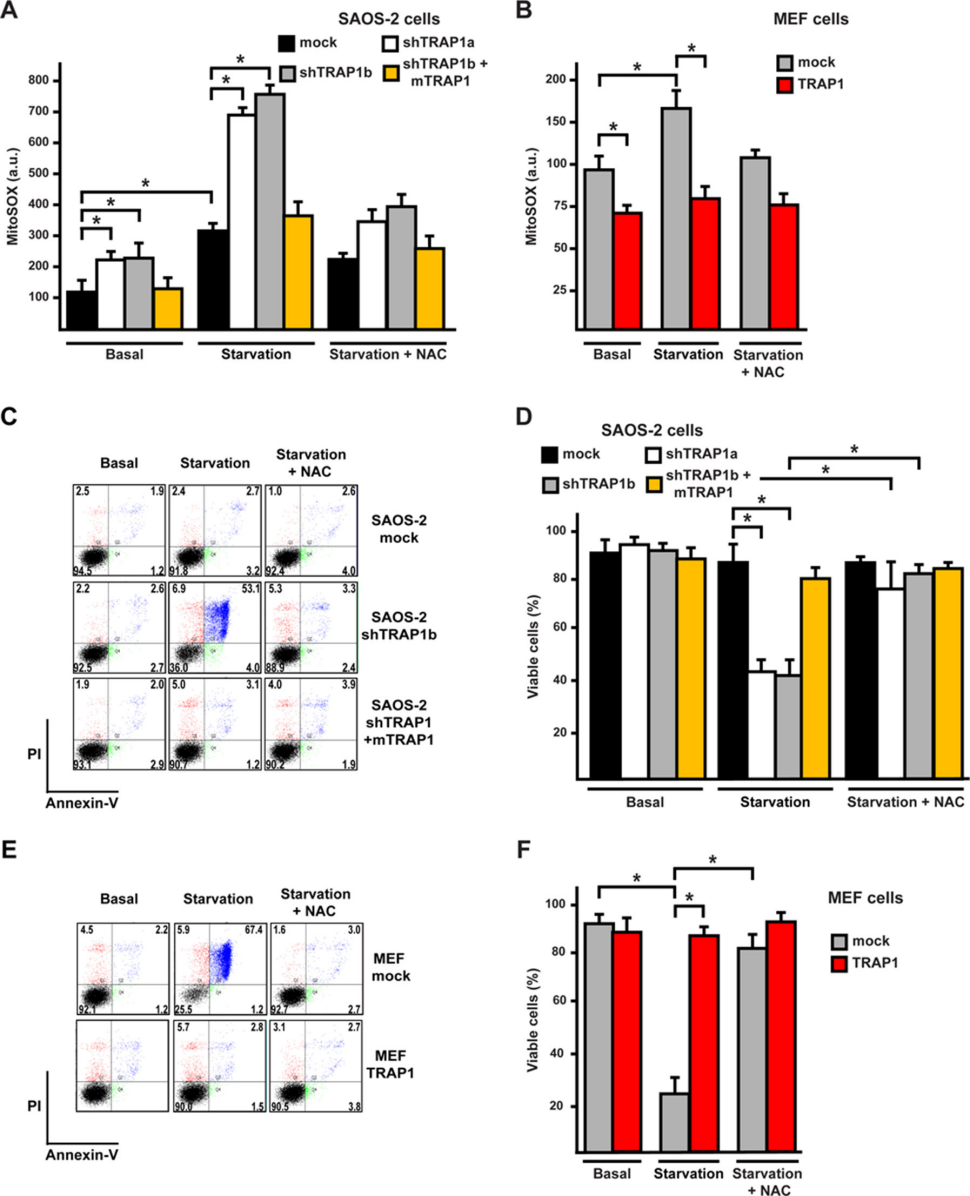
TRAP1 expression protects cells from oxidative stress and death elicited by serum and glucose depletion **(A, B)** Cytofluorimetric analysis of mitochondrial superoxide levels was carried out by staining SAOS-2 (A) or MEF (B) cells with the fluorescent probe MitoSox. Cells were either kept in complete medium (Basal) or depleted of serum and glucose for 8 hours. **(C-F)** Cytofluorimetric analysis of SAOS-2 (C, D) or MEF (E, F) cell viability in complete medium or after 24 hours of serum and glucose depletion. In (C, E) representative traces of cytofluorimetric cell death analysis by Annexin-V and propidium iodide staining are reported. In black, double negative, viable cells; in green, Annexin-V positive, apoptotic cells; in blue, Annexin-V/PI double positive, late apoptotic cells; in red, PI positive, necrotic cells. The percentage of each subpopulation of cells is reported. Where indicated, cells were incubated in the presence of N-acetyl cysteine (NAC, 1 mM). In (D, F) bars indicate percentages of viable, Annexin-V and propidium iodide negative, cells. All along the Figure, SAOS-2 and MEF cells are indicated as in Figure 1, and data are reported as mean±SD values (n≥3; *P<0.05 in a Student's *t* test).

To evaluate whether modulation of ROS levels by TRAP1 might have an impact on cell viability, we placed cells in a medium containing pyruvate and glutamine, but devoid of serum and glucose. Under these conditions ROS levels are strongly increased, as cells cannot use glucose to maintain their redox equilibrium through the pentose phosphate pathway, and are forced to boost oxidative phosphorylation (OXPHOS), thus enhancing ROS production by respiratory chain complexes while decreasing antioxidant defenses [30]. Indeed, serum and glucose depletion augmented mitochondrial superoxide levels, and this pro-oxidant effect was markedly increased in shTRAP1 cells and inhibited by N-acetyl-cysteine (NAC) (Fig. 2A, S1A). The rise in ROS caused by starvation in shTRAP1 cells was paralleled by cell death induction, and this was abrogated by NAC (Fig. 2C, 2D and S1B). Accordingly, both the upsurge in superoxide levels (Fig. 2B) and cell death prompted by starvation (Fig. 2E, 2F) were blocked by TRAP1 overexpression in MEF cells, or by expression of the shRNA-insensitive mTRAP1 construct in SAOS-2 cells (Fig. 2A, 2C, 2D).

### SDH inhibition by TRAP1 shields from oxidative stress

**Figure 3:**
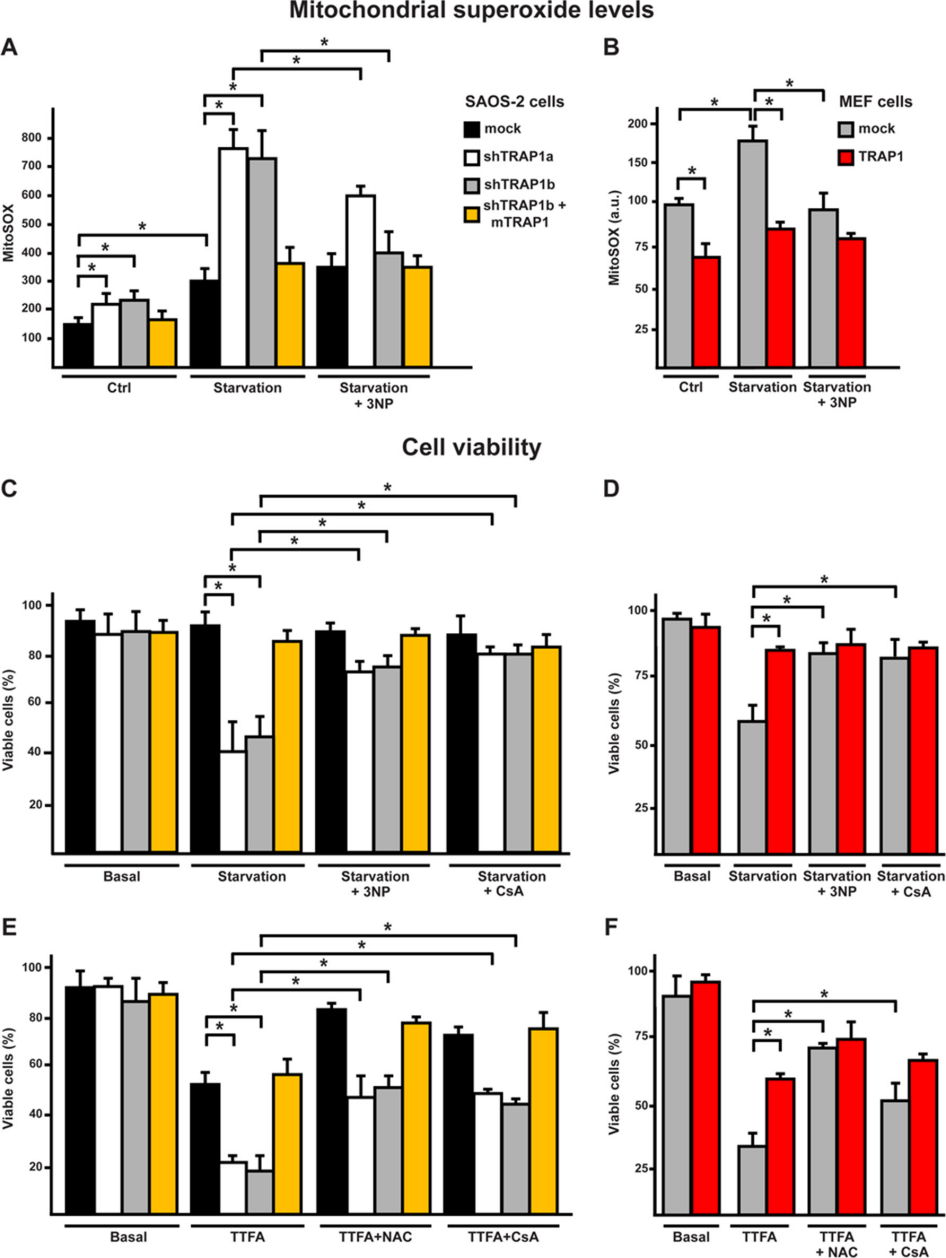
Starvation induces a SDH-dependent oxidative stress which leads to cell death in shTRAP1 cells **(A, B)** Cytofluorimetric measurements of mitochondrial superoxide levels with the MitoSOX probe in SAOS-2 (A) or MEF (B) cells kept for 8 hours without serum and glucose. **(C, D)** Cytofluorimetric viability analysis of SAOS-2 (C) or MEF (D) cells after 24 hours of serum and glucose depletion. Where indicated, cells were incubated in the presence of the SDH inhibitor 3-nitro propionic acid (3-NP, 500 μM) or of the PTP inhibitor cyclosporine A (CsA, 1.6 μM). **(E)** Viability analysis of SAOS-2 cells treated with the SDH inhibitor thenoyltrifluoroaceton (TTFA, 1 mM) for 1 hour. Where indicated, cells were preincubated for 40 minutes with CsA (1.6 μM) or NAC (1 mM). In (C-E), bars indicate percentages of viable, Annexin-V and propidium iodide negative, cells. All along the Figure, SAOS-2 and MEF cells are dubbed as in Figure 1, and data are reported as mean±SD values (n≥3; *P<0.05 in a Student's *t* test).

We have recently found [16] that TRAP1 down-modulates the enzymatic activity of SDH. TRAP1 competes with 3-nitropropionic acid (3-NP), a compound that binds to the catalytic site of the A subunit of SDH and abolishes succinate oxidation and ROS generation [31]. Therefore, TRAP1 inhibition of SDH might have an anti-oxidant effect similar to that of 3-NP. We found that concentrations of 3-NP sufficient to selectively decrease respiration in cells expressing low TRAP1 levels, *i.e.* shTRAP1 neoplastic cells and mock-transfected MEF cells, were also effective in blocking the increase in mitochondrial superoxide and in cell death elicited by serum and glucose depletion; notably, 3-NP was ineffective in cells harboring high TRAP1 levels (mock cancer cells and TRAP1-overexpressing MEF cells) undergoing starvation (Fig. 3A-D, S2A, S2B).

Thenoyltrifluoroacetone (TTFA) inhibits SDH activity downstream to the 3-NP site, as it binds to residues at the quinone-binding site of the B and D subunits of SDH. Thus, TTFA arrests electron transfer from succinate to coenzyme Q but, at variance from 3-NP, induces generation of high superoxide anion levels through SDH [32] [31]. We observed that TTFA toxicity was markedly higher in cells with low TRAP1 levels (shTRAP1 cancer cells or mock MEFs), which were protected by NAC (Fig. 3E, 3F, S2C). Thus, it can be envisioned that TRAP1 interaction with the catalytic site of SDH-A can decrease the electron flow to coenzyme Q, inhibiting ROS production at the site targeted by TTFA. Taken together, these experiments indicate that complex II activity strongly contributes to the increase in ROS levels caused by serum and glucose depletion, and that TRAP1 expression shields cells from this oxidative stress and abrogates the generation of superoxide anions by SDH.

### TRAP1 inhibits ROS-dependent opening of the PTP

**Figure 4:**
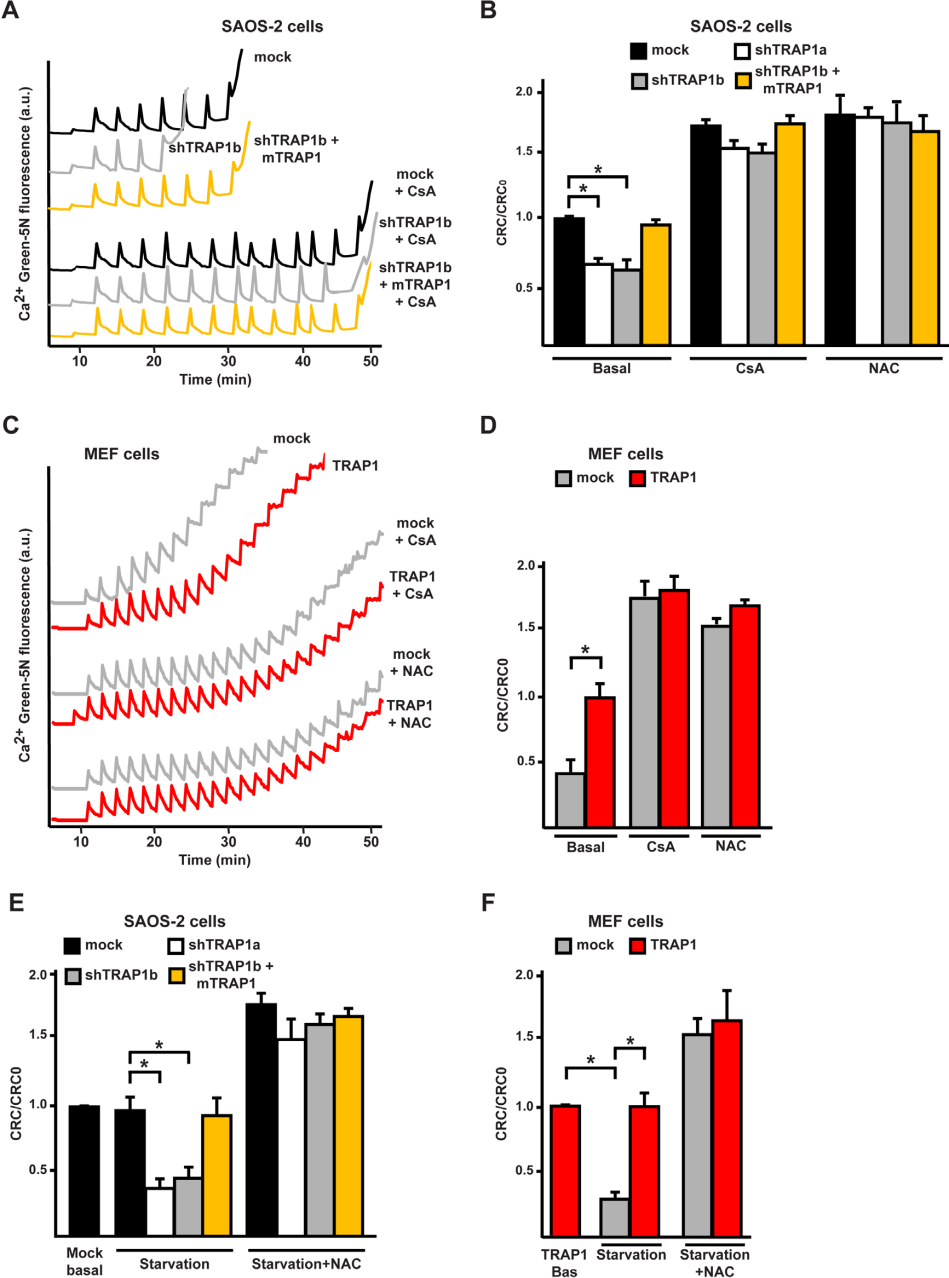
TRAP1 expression protects cells from PTP opening **(A, C)** Representative whole-cell Ca^2+^ retention capacity assays were performed either on SAOS-2 (A) or MEF (C) cells. Fluorescence of Ca^2+^ Green-5N in digitonin-permeabilized cells is reported as arbitrary units on the *y* axis. As the probe does not permeate mitochondria, Ca^2+^ uptake into the organelles after each pulse (5 μM Ca^2+^) causes a rapid decrease of the fluorescence spike. Pore inhibitors and inducers increase or decrease, respectively, the number of spikes before permeability transition, that is revealed by a sudden and marked fluorescence increase, occurs. Where indicated, cyclosporine A (CsA, 1.6 μM) or NAC (1 μM) were added 5 min before starting the assay. **(B, D-F)** bars indicate the ratio between the Ca^2+^ uptake before PTP opening detected in the different experimental conditions (CRC) and in untreated mock SAOS-2 or MEF TRAP1 cells (CRC0). In (E) and (F), CRC of cells undergoing 8 hours of serum and glucose starvation is compared with control conditions. All along the Figure, SAOS-2 and MEF cells are dubbed as in Figure 1, and data are reported as mean±SD values (n≥3; *P<0.05 in a Student's *t* test).

Oxidative stress to mitochondria can induce the PTP, irreversibly committing cells to death [8], and TRAP1 was proposed to inhibit PTP opening [33]. To assess if PTP opening plays a role in the modulation of oxidative insults by TRAP1, cells were deprived of serum and glucose in the presence of cyclosporine A (CsA), which desensitizes pore opening by inhibiting the PTP regulator cyclophilin D (CyP-D) [7]. We found that death induction was abrogated by CsA in cells with low TRAP1 levels (shTRAP1 cancer cells or mock MEFs; Fig. 3C, 3D, S2B). To investigate whether the anti-oxidant activity of TRAP1 protects cells from death by inhibiting PTP opening, we performed a whole-cell Ca^2+^ retention capacity assay, which allows a quantitative assessment of PTP induction [34]. TRAP1 protein levels were inversely correlated to PTP opening; indeed, knocking-down TRAP1 expression increased PTP sensitivity to Ca^2+^ in cancer cells (Fig. 4A, 4B, S3A), whereas TRAP1 overexpression inhibited pore opening in MEF cells (Fig. 4C, 4D). Notably, PTP opening was inhibited not only by CsA, as expected, but also by NAC, in accord with the role of TRAP1-dependent ROS modulation in PTP regulation (Fig. 4A-D, S3A). PTP was further induced in shTRAP1 cells undergoing starvation with respect to cells kept in complete medium; re-expression of mTRAP1, TRAP1 overexpression or treatment with NAC protected from PTP opening in starved cells (Fig. 4E, 4F, S3B). Moreover, when cells were treated with EM20-25, a PTP inducer [35] that boosts superoxide anion generation by respiratory complex I [36], the pore was sensitized in shTRAP1 cells (Fig. S4A, S4D), which were selectively killed by EM20-25 in a NAC- and CsA-sensitive way (Fig. S4B, S4C, S4E).

### TRAP1 promotes *in vitro* tumorigenic growth by protecting cells from oxidative insults

**Figure 5:**
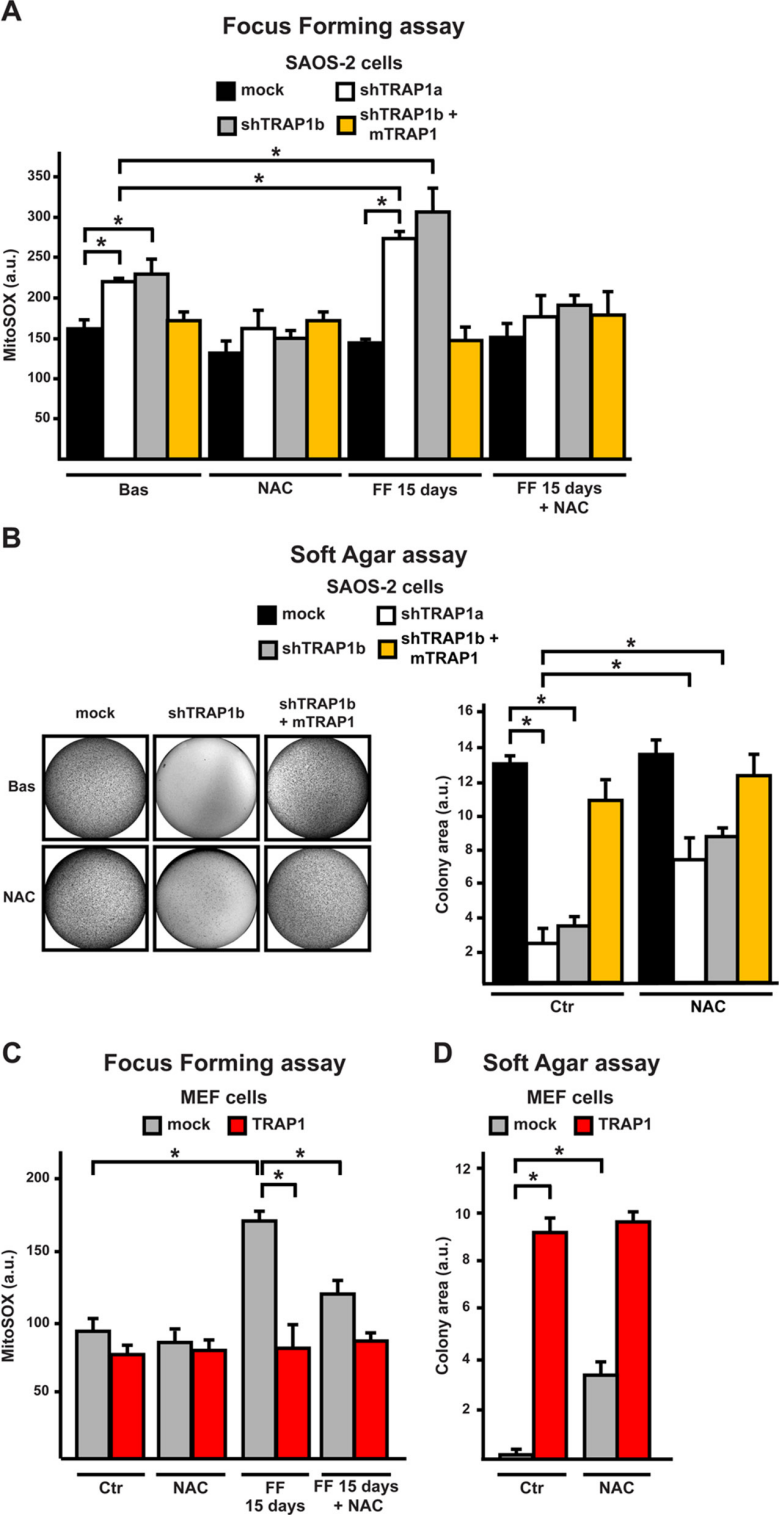
ROS inhibition enhances tumorigenicity in cells with low TRAP1 expression levels **(A, C)** Cytofluorimetric measurements of mitochondrial superoxide levels with the MitoSOX probe in SAOS-2 (A) or MEF (C) cells. Measurements were performed either in standard culture conditions (Basal) or after 15 days of a focus forming assay, *i.e.* 1–2 days before SAOS-2 shTRAP1 and MEF mock cells undergo plate detachment and massive death, without forming any foci. **(B, D)** Soft agar assays in SAOS-2 or MEF cells. Bar graphs indicate the total colony area at the 20^th^ experimental day. Where indicated, experiments were carried out in the presence of NAC (2.5 mM). All along the Figure, SAOS-2 and MEF cells are dubbed as in Figure 1, and data are reported as mean±SD values (n≥3; *P<0.05 in a Student's *t* test).

We reasoned that TRAP1-dependent protection from oxidative insults might contribute to neoplastic growth. Indeed, measurements of mitochondrial superoxide anion levels in cells undergoing a focus forming transformation assay revealed that mitochondrial ROS levels increased only in shTRAP1 cells: this increase was NAC-sensitive and became evident at the 15^th^ day, 1–2 days before these cells underwent massive death (Fig. 5A, S5A). In the mirror experiment, TRAP1 overexpression in MEF cells abolished the ROS surge during the focus forming assay (Fig. 5C). Notably, cells with low TRAP1 levels (shTRAP1 cancer cells or mock MEFs) were unable to form colonies in soft agar; anti-oxidants (both NAC and Trolox) rescued their tumorigenic potential, while they could not further increase colonies in TRAP1-expressing cells (Fig. 5B, 5D, S5B-E).

Taken together, these results indicate that TRAP1 down-modulation of ETC complex II inhibits mitochondrial superoxide production, and renders cells refractory to oxidative stress and PTP opening and prone to transformation.

## DISCUSSION

To fuel growth and proliferation, neoplastic cells orchestrate dramatic metabolic rearrangements [37]. The ensuing induction of anabolic activities, together with respiratory inhibition and fluctuations of oxygen supply in the tumor microenvironment, exposes neoplastic cells to the risk of oxidative insults, forcing them to the tight maintenance of an appropriate redox equilibrium [1, 2]. In this scenario, mitochondria play a central role, as they integrate a variety of signals to finely tune cellular energy state and metabolite concentrations, and utilize sublethal concentrations of ROS in order to regulate diverse biological routines such as response to stress or cell survival, proliferation, metabolic homeostasis and motility. Therefore, changes in respiratory levels that occur during the neoplastic process largely impact on tumor growth, as respiratory complexes are a primary source of ROS generation [4, 38]. Mitochondria can also actively take part in tumorigenesis by modulating the amount of some metabolites, such as 2-hydroxy-glutarate, fumarate, and succinate, which accumulate following mutations of TCA cycle enzymes and have been termed oncometabolites for their pro-neoplastic roles [39]. However, mutations leading to oncometabolite accumulation are restricted to specific tumor subsets, which makes their general neoplastic role less clear.

Our findings place the mitochondrial chaperone TRAP1 at the crossroad of mitochondrial signalling in tumor cells. TRAP1 interacts with SDH, down-modulating its enzymatic activity and increasing intracellular levels of the oncometabolite succinate. In turn, succinate inhibits the prolyl hydroxylase that primes HIF1α for proteasomal degradation, and HIF1α stabilization is required for tumorigenesis both *in vitro* and in xenografts of TRAP1-expressing tumor cells [16]. Moreover, here we demonstrate that TRAP1 interaction with SDH contributes to minimize ROS production by SDH, shielding neoplastic cells from mitochondrial PTP opening and apoptosis.

To explain these effects of TRAP1, we propose a model in which TRAP1 decreases succinate:coenzyme Q reductase activity by perturbing succinate interaction with the catalytic site of SDHA subunit. Consistent with this hypothesis, we have previously shown that a very high concentration of 3-NP, an inhibitor of succinate oxidation at the catalytic site of SDHA subunit [31], is required to block SDH activity in TRAP1-expressing cells [16], which suggests that TRAP1 interferes with 3-NP interaction at the SDHA catalytic site. The inhibitory interaction of TRAP1 with SDHA would both hinder succinate oxidation, inducing accumulation of the metabolite, and lower electron funneling through other SDH subunits, inhibiting ROS generation caused by electron leak and explaining the anti-oxidant effect of TRAP1. Accordingly, here we observe that a low concentration of 3-NP down-modulates the amount of mitochondrial superoxide anion in cells expressing low TRAP1 levels (either shTRAP1 cancer cells, or mock MEF cells), whereas it is not effective in cells with high TRAP1 levels. Moreover, we show that cells with low TRAP1 levels are sensitized to oxidative stress induced by TTFA, which generates ROS at complex II downstream to the 3-NP/TRAP1 site, as it blocks electron transfer to coenzyme Q at the quinone-binding site [31]. In our model, TRAP1 inhibits oxidative stress by TTFA as it produces an upstream bottleneck in the electron flux by inhibiting succinate oxidation.

Notably, cells expressing low TRAP1 levels are sensitized to conditions of starvation, as these cause a marked ROS increase and elicit apoptosis, which is abrogated by 3-NP or by inhibiting PTP opening and is not detected in TRAP1-expressing cells. Therefore, inhibition of SDH by TRAP1 contributes to the buildup of anti-oxidant defenses in conditions of nutrient depletion that mimic those faced by cancer cells during tumor growth. By preventing the massive death caused by ROS-dependent opening of the mitochondrial PTP, TRAP1 might favor the primary accretion of the tumor, and indeed we observe that mitochondrial superoxide levels increase during the focus forming assay in cells with low TRAP1 levels, and that ROS scavengers rescue the *in vitro* tumorigenicity of shTRAP1 cells. At the same time, by stabilizing HIF1 in a succinate-dependent fashion, TRAP1 sets a pseudohypoxic phenotype that prepares neoplastic cells to stand conditions of oxygen shortage before these occur, and that can increase malignancy by triggering HIF1-driven epithelial mesenchymal transition, angiogenesis and further metabolic adaptations [40, 41]. An inverse correlation between TRAP1 expression and tumor grade was also observed, suggesting that the absence of TRAP1 might favor ROS-dependent tumor invasiveness, possibly enhancing activity of respiratory complex IV [42]. This is in accord with a model by which the absence of TRAP1 would prompt an increase in ROS levels at later tumor stages, contributing to a more aggressive phenotype by increasing cell motility and genomic instability of the neoplasm [15]. However, further work is needed to better clarify the role(s) of TRAP1 in tumorigenesis, as it was also reported that its levels increase during some form of malignant progression, as in prostate cancer or colorectal carcinoma [15].

Notably, both succinate-dependent stabilization of HIF1, and modulation of intracellular redox equilibrium have been proposed as oncogenic factors in SDH-deficient cancers [18]. While succinate accumulation is an important feature of these tumors, it must be highlighted that loss-of function oncogenic mutations of SDHA subunit are extremely rare. The much more common SDHB-D mutations could be related to increased intracellular ROS levels, but this issue remains controversial [19, 20]. However, the partial and reversible SDH inhibition caused by TRAP1 is different from the complete block of the SDH enzyme that follows its loss-of-function mutations. Moreover, TRAP1 is a phosphorylation target of the Ser/Thr kinase PINK1 [26] and of the Tyr kinase Src [42], and it is reported to be both phosphorylated and acetylated in public databases [15]; therefore, it can be envisioned that post-translational modifications might further affect TRAP1 modulation of SDH enzymatic activity. Indeed, this dynamic interaction between TRAP1 and SDH could provide a very flexible response to fit the redox equilibrium and the pseudohypoxic status required for neoplastic progression, acting as a general oncogenic mechanism, whereas SDH mutations remain confined to very specific tumor subsets.

As it was shown by us and others that TRAP1 interacts with respiratory complex IV [16, 42], we cannot exclude that other interactions of TRAP1 with OXPHOS components might contribute to modulate respiration and ROS generation. The similarity of TRAP1 and Hsp90, a chaperone characterized by a huge number of client proteins [43], further suggests the possibility of multiple interactions. Nevertheless, SDH inhibition affects respiration both directly, by decreasing electron flux to respiratory complex III; and indirectly, as SDH is part of the TCA cycle and provides reducing equivalents to fuel respiration. Thus, the effect of TRAP1 on SDH could extend to a more general down-modulation of respiration, affecting ROS generation at the level of several respiratory complexes.

Several pieces of evidence point towards a role of the mitochondrial PTP as cell death effector in cells with low TRAP1 levels. In accord with observations indicating that thiol oxidation sensitizes the PTP [6], low TRAP1 levels sensitize cells to pore opening, both in basal conditions and after starvation or treatment with EM20-25, a PTP inducer that prompts a massive ROS surge; and PTP desensitization with CsA protects them from apoptosis. PTP desensitization is emerging as an important strategy through which cancer cells escape death [10], and we have observed that the chaperone CyP-D, an important PTP modulator, is inhibited by an ERK/GSK3 signal transduction axis in tumor cells [9]. TRAP1 was shown to prevent PTP opening both in hypoxic cardiomyocytes [28] and in neoplastic cell models, where it interacts with CyP-D [33]. These data suggest that TRAP1 could take part in a multi-chaperone mitochondrial complex that keeps the pore locked in tumor cells, possibly through multiple mechanisms, thus favoring the apoptosis evasion that hallmarks them [44].

The pleiotropic biological activities of TRAP1 are emerging as crucial features in the process of neoplastic transformation, which warrants a deeper comprehension of TRAP1 biochemistry. For instance, functional dynamics studies of structural modifications occurring during TRAP1 chaperone cycle could lead to the identification of druggable sites, in analogy with what has been done for Hsp90 [45, 46]; thus, selectively targeting TRAP1 chaperone activity might be a promising strategy for the development of novel antineoplastic drugs.

## METHODS

### Cell culture

Human SAOS-2 osteosarcoma cells and human HeLa cervix carcinoma cells were purchased from ATCC. MEF cells were obtained from C57BL/6J mice through SV40-immortalization. All cells were grown in Dulbecco's modified Eagle's medium (DMEM) supplemented with 10% fetal bovine serum (Invitrogen), 100 units/ml penicillin and 100 μg/ml streptomycin, in a humidified atmosphere of 5% CO_2_/95% air at 37°C. TRAP1 stable interference of SAOS-2 and HeLa cells was achieved by transfecting cells with the following TRAP1 shRNAs (Sigma): a) CCGGCAGAGCACTCACCCTACTAT GCTCGAGCATAGTAGGGTGAGTGCTCTGTTTTTG; b) CCGGGCGCTCATCAAGAAGCTGAATCTCGAGA TTCAGCTTCTTGATGAGCGCTTTTTG; stable interfered cells were selected in 0.8 μg/ml puromycin (Sigma). A mouse TRAP1 cDNA (Origene) was expressed in shTRAP1 cells obtained with sequence b), which is highly divergent between human and mouse genes. MEF cells were transfected with a TRAP1 cDNA (cloned in a pCMV6 vector, Origene), and selected in G418 (0.5 mg/ml; Sigma).

### Western immunoblot assays

For Western immunoblots analyses, cells were lysed at 4°C in a buffer composed by 140 mM NaCl, 20 mM Tris-HCl pH 7.4, 5 mM EDTA, 10% glycerol, 1% Triton X-100, in the presence of phosphatase and protease inhibitors (Sigma). Lysates were then cleared with a centrifugation at 13000*g* for 30 min at 4°C, and proteins were quantified using a BCA Protein Assay Kit (Thermo Scientific-Pierce). Proteins were then separated under reducing conditions on SDS-polyacrylamide gels, transferred onto Hybond-C Extra membranes (Amersham) and visualized by enhanced chemiluminescence (Millipore) following standard methods. Anti TRAP1 mouse monoclonal antibodies were from Santa Cruz (sc-13557) and from Becton Dickinson (612344); anti GAPDH (MAB374) mouse monoclonal antibody was from Chemicon.

### Cell viability assays

Cell viability was assessed with flow cytometry recordings as described [47]. Briefly, cells were stained with FITC-conjugated Annexin-V (Boehringer Mannheim) and propidium iodide (PI, 1 μg/ml; Sigma), to determine phosphatidyl-serine exposure on the cell surface (increased FITC-conjugated Annexin-V staining) and loss of plasma membrane integrity (PI permeability and staining). Cells were incubated at 37°C in 135 mM NaCl, 10 mM HEPES, 5 mM CaCl_2_ and then analyzed on a FACS Canto II flow cytometer (Becton Dickinson). Data acquisition and analysis were performed using FACSDiva software.

### ROS measurements

Intracellular hydrogen peroxide levels were determined with the use of N-acetyl-3, 7-dihydroxyphenoxazine (Amplex Red, Invitrogen), following manufacturer's instructions, in 50–100 μg of fresh cell lysates. Mitochondrial superoxide levels were assessed by cytofluorimetric analyses with the use of the fluorescent probe MitoSOX (1 μM; Invitrogen).

### Whole-cell Ca^2+^ retention capacity (CRC) assays

CRC assays were performed as described [9, 34] to assess PTP opening in whole cells exposed to Ca^2+^ pulses. Cells were washed in an isotonic buffer (130 mM KCl, 1 mM Pi-Tris, 10 mM Tris/Mops, and 0.1 mM EGTA/Tris, pH 7.4) and permeabilized with 100 μM digitonin (10 min, 4°C) after increasing to 1 mM EGTA concentration. Digitonin was then eliminated and permeabilized cells were placed in low (10 μM) EGTA in the presence of (5 mM Glutamate/2.5 mM Malate) 2 μM rotenone/5 mM succinate, 10 μM cytochrome *c*, and of the Ca^2+^ probe Calcium Green-5N (0.5 μM; λ exc: 505 nm; λ em: 535 nm; Molecular Probes), which does not permeate mitochondria. Cells were then exposed to trains of Ca^2+^ pulses (5 μM each); the subsequent fluorescence increases lasted until Ca^2+^ was taken up by mitochondria, leading to a rapid signal drop. PTP opening was detected as a sudden and irreversible fluorescence rise. Ca^2+^ taken up by mitochondria before PTP opening in the different experimental conditions was normalized to control conditions (indicated as CRC_0_). Experiments were performed with a Fluoroskan Ascent FL (Thermo Electron Corp.) plate reader.

### Tumorigenesis assays

Two different *in vitro* tumorigenesis assays were performed: (1) focus forming assays, in which tumorigenicity is assessed by the capability of cells to overgrow confluence and form foci. For these experiments, 10^6^ cells were plated in 10 cm Petri dishes (BD Falcon) in Dulbecco's modified Eagle's (DMEM) medium supplemented with 10% fetal bovine serum (Gibco). When cells reached sub-confluence, serum concentration was decreased to 0.5% and changed every 4^th^ day. At the 15^th^ day after serum decrease cells were utilized for the cytofluorimetric assessment of mitochondrial superoxide levels with MitoSox; (2) soft agar assays, in which tumorigenicity is assessed by the capability of cells to form colonies when placed in an agar matrix. For these experiments, cells (3×10^5^) were embedded in DMEM medium supplemented with 0.5% serum and mixed with low melting point agarose at a final concentration of 0.6%, and placed on a bottom layer composed by DMEM medium mixed with low melting point agarose (Promega) at a final concentration of 1.0%. Fresh medium (DMEM 0.5% serum) was added on the top of the two layers every 4^th^ day. At the 20^th^ day, dishes were washed in PBS and colonies were stained with Crystal Violet 0.005% and analyzed with Image Analyzer custom software [48].

## SUPPLEMENTARY FIGURES


