# Supplementary material for: Inhibition of succinate dehydrogenase by the mitochondrial chaperone TRAP1 has anti-oxidant and anti-apoptotic effects on tumor cells
Source: Oncotarget. 2014 Nov 15;5(23):11897–908. doi: 10.18632/oncotarget.2472 (PMC4323003; doi:10.18632/oncotarget.2472)
Supplement: Supplementary file 1 [file oncotarget-05-11897-s001.pdf]

SUPPLEMENTARY FIGURES

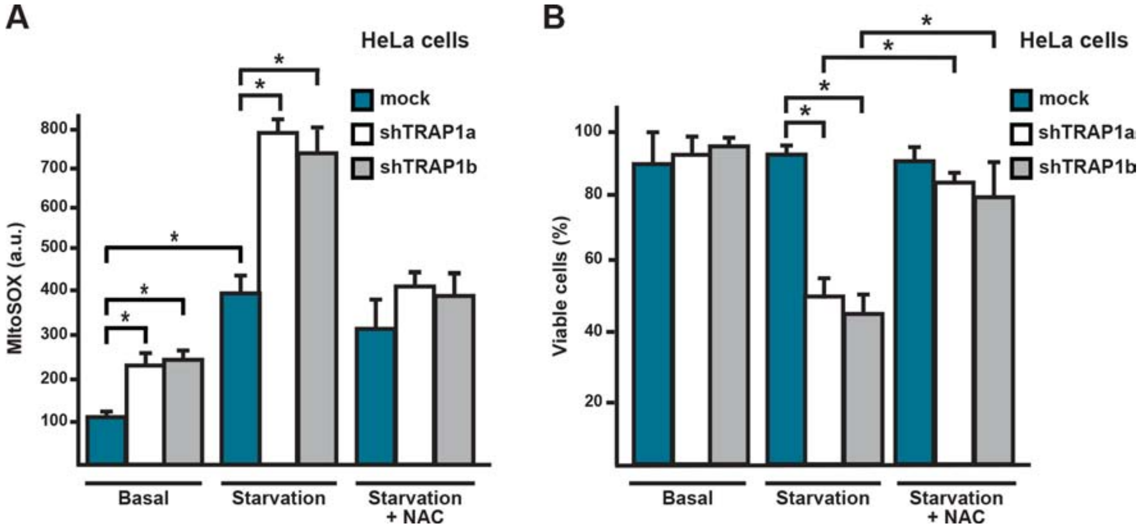

Supplementary Figure S1: TRAP1 expression protects HeLa cells from oxidative stress and death elicited by serum and glucose depletion. (A) Cytofluorimetric analysis of mitochondrial superoxide levels was carried out by staining HeLa cells with the fluorescent probe MitoSox. Cells were either kept in complete medium (Basal) or depleted of serum and glucose for 8 hours. **(B)** Cytofluorimetric analysis of cell viability in complete medium or after 24 hours of serum and glucose depletion. Bars indicate percentages of viable, Annexin-V and propidium iodide negative, cells. Where indicated, cells were incubated in the presence of N-acetyl cysteine (NAC, 1 mM). All along the Figure, HeLa cells are indicated as in Figure 1, and data are reported as mean±SD values (n≥3; *P<0.05 in a Student's *t* test).

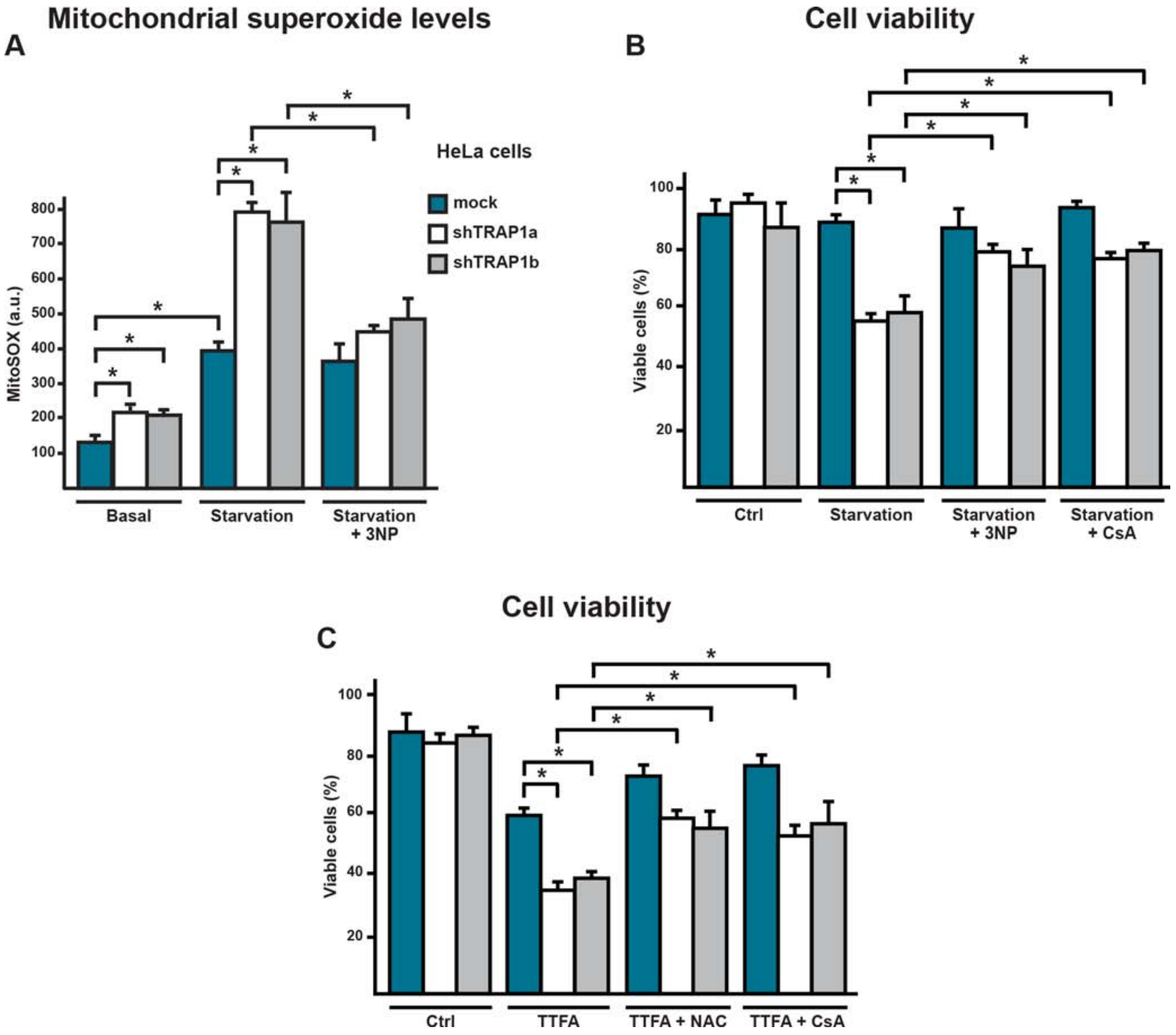

Supplementary Figure S2: Starvation induces a SDH-dependent oxidative stress which leads to cell death in HeLa shTRAP1 cells. **(A)** Cytofluorimetric measurements of mitochondrial superoxide levels with the MitoSOX probe in HeLa cells kept for 8 hours without serum and glucose. **(B)** Cytofluorimetric viability analysis of HeLa cells after 24 hours of serum and glucose depletion. Where indicated, cells were incubated in the presence of the SDH inhibitor 3-nitro propionic acid (3-NP, 500 µM) or of the PTP inhibitor cyclosporine A (CsA, 1.6 µM). **(C)** Viability analysis of HeLa cells treated with the SDH inhibitor thenoyltrifluoroacetone (TTFA, 1 mM) for 1 hour. Where indicated, cells were preincubated for 40 minutes with CsA (1.6 µM) or NAC (1 mM). In (B-C), bars indicate percentages of viable, Annexin-V and propidium iodide negative, cells. All along the Figure, HeLa cells are dubbed as in Figure 1, and data are reported as mean±SD values ($n \geq 3$; *$P<0.05$ in a Student's *t* test).

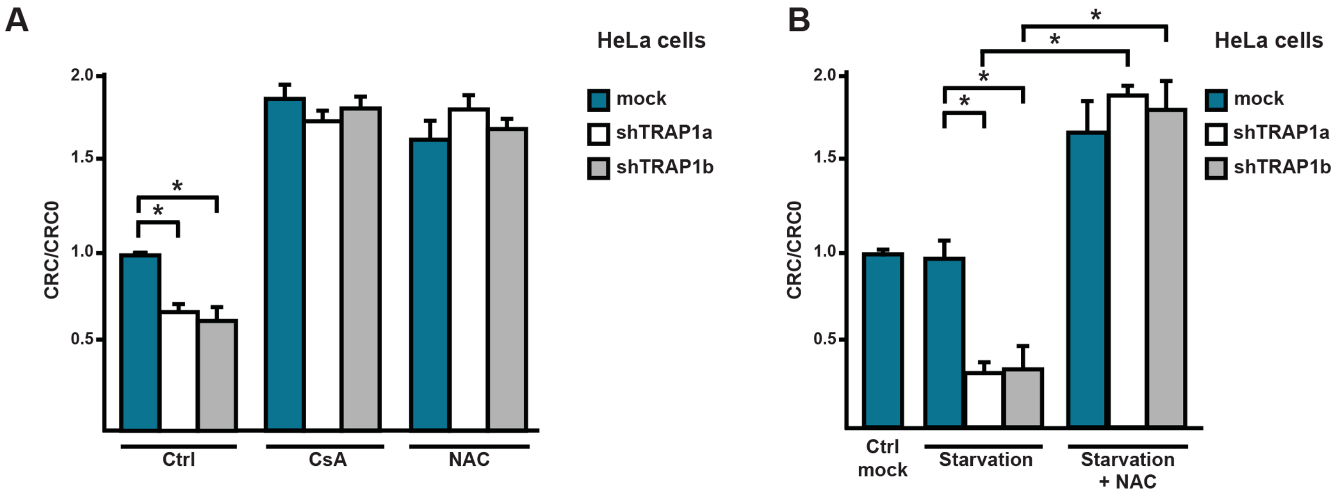

Supplementary Figure S3: TRAP1 expression protects HeLa cells from PTP opening. (A) Whole-cell CRC assay. Bars indicate the ratio between the Ca2+ uptake before PTP opening detected in the different experimental conditions (CRC) and in untreated mock HeLa cells (CRC0). **(B)** CRC of cells undergoing 8 hours of serum and glucose starvation is compared with control conditions. Where indicated, cyclosporine A (CsA, 1.6 µM) or NAC (1 mM) were added 5 min before starting the assay. All along the Figure, HeLa cells are dubbed as in Figure 1, and data are reported as mean±SD values (n≥3; *P<0.05 in a Student's *t* test)

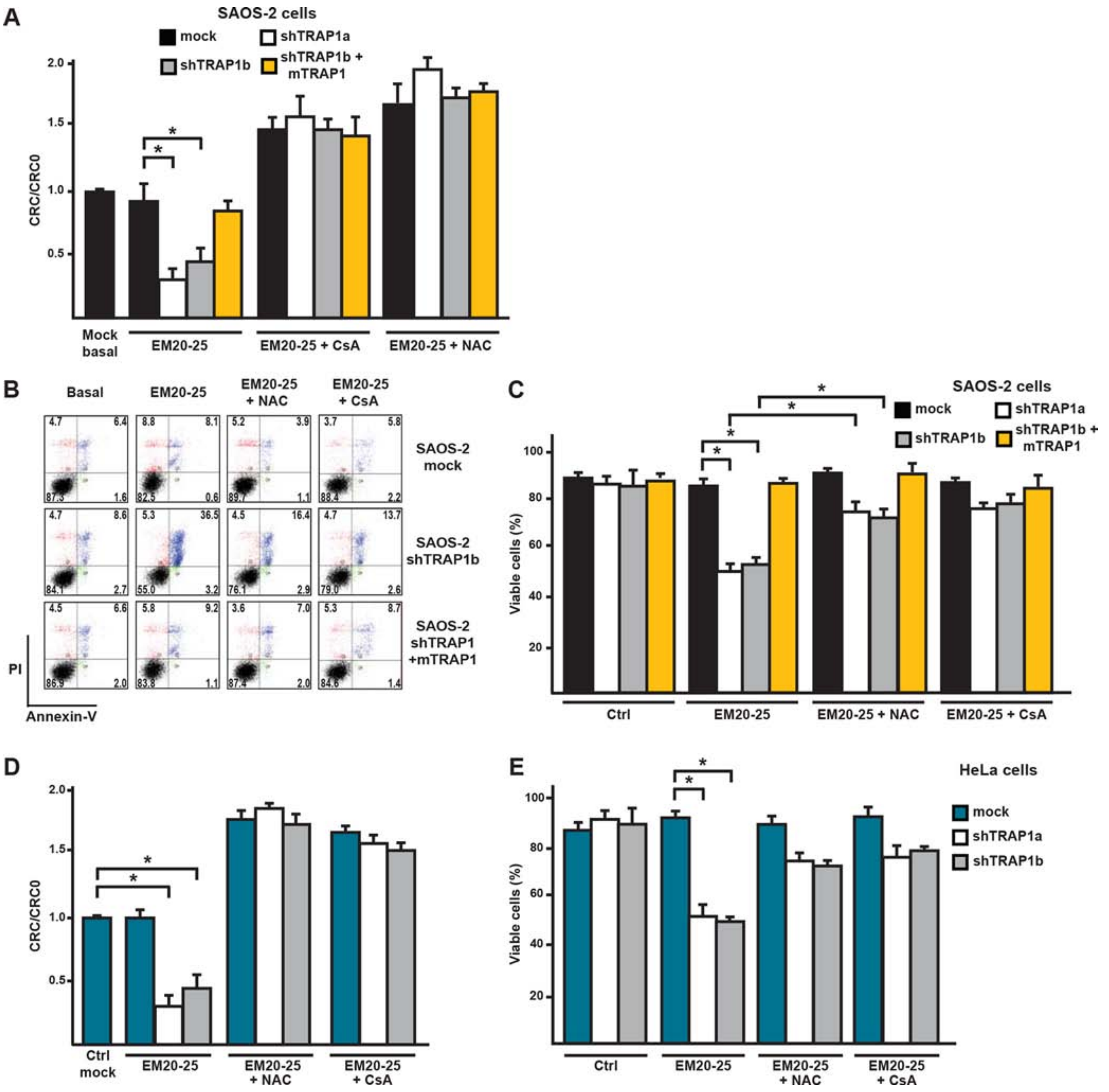

Supplementary Figure S4: TRAP1 expression protects cells from oxidative insults and cell death elicited by the PTP inducer EM20-25. (A, D) Whole-cell CRC assay in SAOS-2 (A) and HeLa (D) cells. Bars indicate the ratio between the Ca^{2}+ uptake before PTP opening detected in the different experimental conditions (CRC) and in untreated mock cells (CRC0). Where indicated, cells were exposed for 2 h to EM20-25 (50 µM); cyclosporine A (CsA, 1.6 µM) or NAC (1 mM) were added 5 min before starting the assay. **(B, C, E)** Cytofluorimetric analysis of SAOS-2 (B, C) or HeLa (E) cell viability after a 2 hour exposure to EM20-25 (500 µM). In (B) representative traces of cytofluorimetric cell death analysis by Annexin-V and propidium iodide staining are reported. Subpopulations of cells are indicated as in Figure 2C. Where indicated, cells were preincubated for 30 min with NAC (1 mM) or with CsA (1.6 µM). In (B, E) bars indicate percentages of viable, Annexin-V and propidium iodide negative, cells. All along the Figure, SAOS-2 and HeLa cells are indicated as in Figure 1, and data are reported as mean±SD values (n≥3; *P<0.05 in a Student's t test).

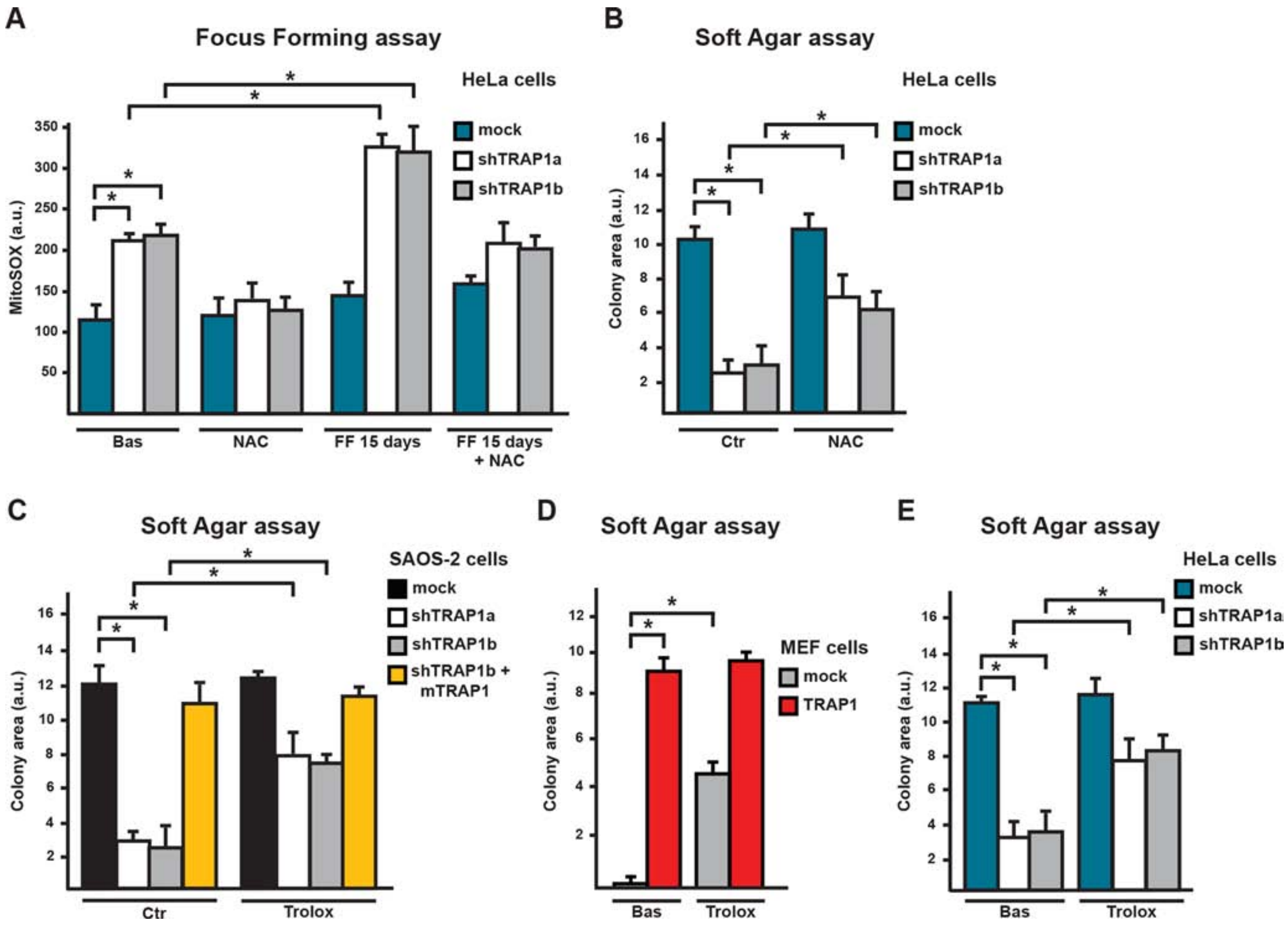

Supplementary Figure S5: ROS inhibition enhances tumorigenicity in cells with low TRAP1 expression levels. (A) Cytofluorimetric measurements of mitochondrial superoxide levels with the MitoSOX probe in HeLa cells were performed as in Figure 5A. **(B-E)** Soft agar. Bar graphs indicate the total colony area at the 20th experimental day. Where indicated, experiments were carried out in the presence of NAC (2.5 mM) or Trolox (100 µM). All along the Figure, cells are dubbed as in Figure 1, and data are reported as mean±SD values ($n \geq 3$; $^*P<0.05$ in a Student's t test).
